# Crystal structure, DFT and Hirshfeld surface analysis of (*E*)-*N*′-[(1-chloro-3,4-di­hydro­naph­thal­en-2-yl)methyl­idene]benzohydrazide monohydrate

**DOI:** 10.1107/S2056989019017183

**Published:** 2020-01-03

**Authors:** H. A. Arjun, G. N. Anil Kumar, R. Elancheran, S. Kabilan

**Affiliations:** a Drug Discovery Lab, Department of Chemistry, Annamalai University, Chidambaram 608002, Tamil Nadu, India; b Department of Physics, Ramaiah Institute of Technology, Bengaluru 560054, India

**Keywords:** crystal structure, benzohydrazide derivative, Hirshfeld surface analysis

## Abstract

In the title compound, C_18_H_15_ClN_2_O·H_2_O, a benzohydrazide derivative, the dihedral angle between the mean plane of the di­hydro­naphthalene ring system and the phenyl ring is 17.1 (2)°. In the crystal, O—H⋯O, N—H⋯O and C—H⋯O hydrogen bonds link the benzohydrazide and water mol­ecules, forming a layer parallel to the *bc* plane. Hirshfeld surface analysis and two-dimensional fingerprint plots indicate that the most important contributions to the crystal packing are from H⋯H (45.7%) and H⋯C/C⋯H (20.2%) contacts.

## Abstract 2

In the title compound, C_18_H_15_ClN_2_O·H_2_O, the dihedral angle between the mean plane of the di­hydro­naphthalene ring system and the phenyl ring is 17.1 (2)°. In the crystal, mol­ecules are linked by O—H⋯O, N—H⋯O and C—H⋯O hydrogen bonds.

## Chemical context   

Benzohydrazides are versatile compounds in medicinal chemistry that are used for the development of new drugs (Veeramanikandan *et al.*, 2015 ▸). Benzohydrazide derivatives are potent inhibitors of prostate cancer (Arjun *et al.*, 2019 ▸) and show anti-inflammatory (Todeschini *et al.*, 1998 ▸), anti-malarial (Melnyk *et al.*, 2006 ▸), entamoeba histolyica (Inam *et al.*, 2016 ▸) and anti-tuberculosis (Bedia *et al.*, 2006 ▸) activities. Herein we describe the mol­ecular and crystal structures of the title compound, which can act as a potential multidrug ligand for various biological activities. The mol­ecular packing was further studied with Hirshfeld surface analysis and PIXEL methods (Sowmya *et al.*, 2018 ▸).
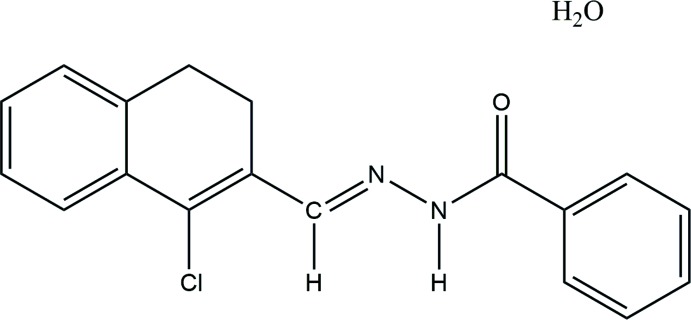


## Structural commentary   

**Figure 1:**
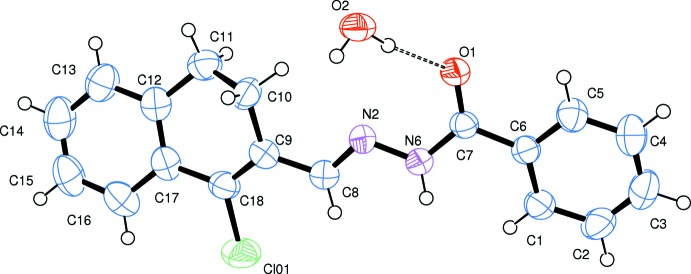
The mol­ecular structure of the title compound, with the atom labelling. Displacement ellipsoids are drawn at the 50% probability level. The O—H⋯O hydrogen bond is indicated by a dashed line.

The mol­ecular structure of the title compound is shown in Fig. 1 ▸. The benzohydrazide mol­ecule adopts an *E* configuration with respect to the C8=N2 bond. The cyclo­hexene ring (C9–C12/C17/C18) adopts nearly a half-chair conformation, as indicated by the total puckering amplitude *Q*_T_ of 0.431 (3) Å and spherical polar angle *θ* = 115.6 (3)° with *φ* = 264.4 (4)°; atom C10 shows a maximum deviation of 0.282 (4) Å from the mean plane. The phenyl ring (C1–C6) and the mean plane of the di­hydro­naphthalene ring system (C9–C18) are inclined to each other by 17.1 (2)°. The central hydrazine fragment (C8/N2/N6/C7/O1) is almost planar, making dihedral angles of 11.0 (2) and 8.49 (18)°, respectively, with the phenyl ring and the mean plane of the di­hydro­naphthalene ring system.

## Supra­molecular features and Hirshfeld surface analysis   

**Figure 2:**
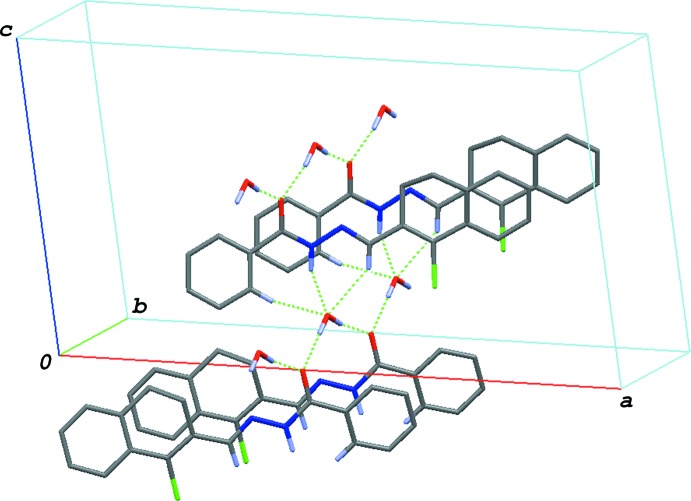
A packing diagram of the title compound, showing the O—H⋯O, N—H⋯O and C—H⋯O hydrogen bonds (dashed lines). H atoms not involved in these inter­actions have been omitted.

In the crystal, the water mol­ecule forms five hydrogen bonds with three benzohydrazide mol­ecules. The benzohydrazide mol­ecules are stacked in a column along the *b-*axis direction through O—H⋯O hydrogen bonds (O2—H2*A*⋯O1^i^ and O2—H2*B*⋯O1; symmetry code as in Table 1 ▸) between the H atoms of the water mol­ecule and the carbonyl O atoms of two adjacent benzohydrazide mol­ecules (Fig. 2 ▸). The water mol­ecule also acts as a hydrogen-bond acceptor from other benzohydrazide mol­ecules: N—H⋯O and C—H⋯O hydrogen bonds (N6—H6⋯O2^ii^, C1—H1⋯O2^ii^ and C8—H8⋯O2^ii^; Table 1 ▸) link the mol­ecules, forming a layer parallel to the *bc* plane.

**Figure 3:**
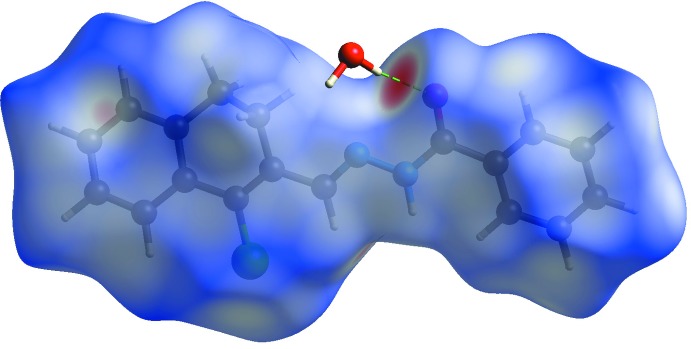
Hirshfeld surface mapped over *d*_norm_ (range −0.575 to 1.326 a.u.) for the title compound showing the O—H⋯O hydrogen bond.

**Figure 4:**
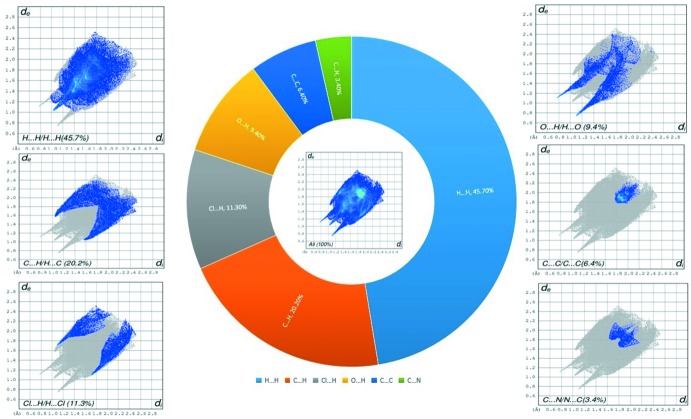
Two-dimensional fingerprint plots for the title compound with the percentage contribution of the inter­molecular contacts. The *d*_i_ and *d*_e_ values are the closest inter­nal and external distances (Å) from given points on the Hirshfeld surface.

Hirshfeld surface analysis was performed using *CrystalExplorer17.5* (Spackman & Jayatilaka, 2009 ▸) to qu­antify and visualize the various inter­molecular contacts in the crystal. The Hirshfeld surface for the title compound mapped over *d*_norm_ is shown in Fig. 3 ▸, where the dark-red spot represents a close contact of the water mol­ecule, corresponding to the O—H⋯O inter­actions. Two-dimensional fingerprint plots are shown in Fig. 4 ▸. The most important contributions to the crystal packing are from H⋯H/H⋯H (45.7%), C⋯H/H⋯C (20.2%), O⋯H/H⋯O (9.4%), Cl⋯H/H⋯Cl (11.3%), C⋯C(6.4%) and C⋯N/N⋯C(3.4%) inter­actions.

## Inter­action energies and theoretical calculations   

**Figure 5:**
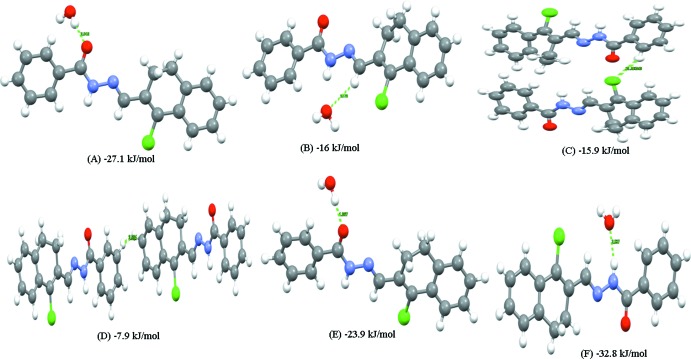
Important mol­ecular pairs in the crystal of the title compound and their inter­action energies.

The various inter­molecular inter­action energies of the title crystal were calculated using the PIXEL-CLP module (Gavezzotti, 2003 ▸). The lattice energy of the crystal structure is found to be −67.2 kJ mol^−1^ with the energy partitioned into Coulombic, polarization, dispersion and repulsion energy components of −68.4, −30.7, −95.3 and 128.1 kJ mol^−1^, respectively. The important mol­ecular pairs (motifs *A*–*F*) and their inter­action energies are shown in Fig. 5 ▸, and the partitioned inter­molecular energies along with the above inter­actions are given in Table 2 ▸. The N—H⋯O inter­action energy in motif *F* (−32.8 kJ mol^−1^) is strongest followed by the O—H⋯O inter­actions in motifs *A* and *E* (−27.1 and −23.9 kJ mol^−1^, respectively), and the C—H⋯O inter­action in motif *B* (−16 kJ mol^−1^).

**Figure 6:**
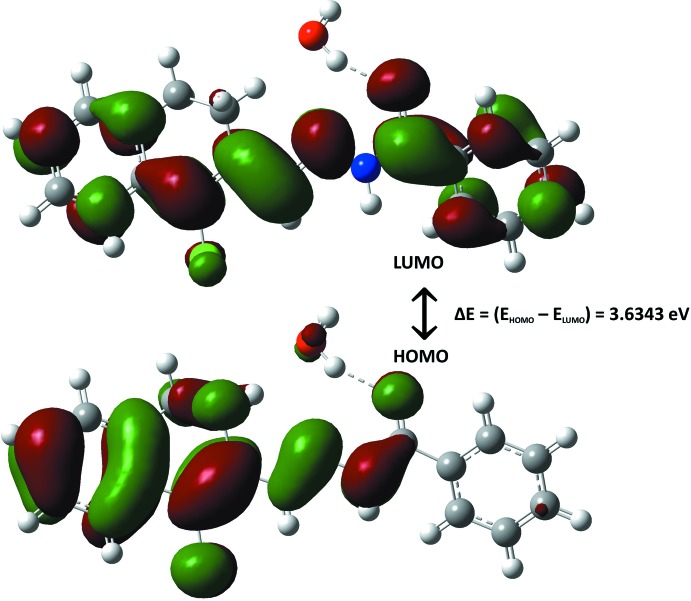
The frontier mol­ecular orbitals, highest-occupied mol­ecular orbital (HOMO) and the lowest-unoccupied mol­ecular orbital (LUMO), calculated for the title compound.

Density functional theory (DFT) calculations using the *B3LYP* (Becke, 1993 ▸) method at the 6-31++G(*d*,*p*) level were performed using *GAUSSIAN09* (Frisch *et al.*, 2009 ▸). The DFT-optimized structure of the title compound is found to be in good agreement with the experimental geometry. Frontier mol­ecular orbitals are plotted to specify the distribution of electronic densities (Fig. 6 ▸); the HOMO–LUMO gap of 3.6349 eV indicates that the nature of mol­ecule is soft. The quantum-chemical parameters, such as hardness (η), softness (ζ), chemical potential (μ), electrophilicity (ω) and electronegativity (χ), were also calculated (Table 3 ▸), using the HOMO and LUMO energies. The electrophilicity index (ω) of 4.3148 eV, which measures the energy lowering due to the electron flow between the donor and acceptor, also supports the soft nature of the title compound. The lower chemical potential (μ) of −3.9602 eV signifies the lesser resistance towards the deformation or polarization of the electron cloud of the atoms or mol­ecule under a small perturbation of chemical reaction.

## Database Survey   

A search of the Cambridge Structural Database (Version 5.39; Groom *et al.*, 2016 ▸) gave 1579 hits for the benzohydrazides with different substituents and 260 hits for their hydrate compounds. The water mol­ecules mediate strong hydrogen bonds in hydrate compounds such as (*E*)-3,4,5-trimeth­oxy-*N*-[(6-meth­oxy-4-oxo-4*H*-chromen-3-yl)methyl­idene]benzo­hydrazide monohydrate (Ishikawa & Watanabe, 2014*a* ▸), (*E*)-4-meth­oxy-*N*-[(6-methyl-4-oxo-4*H*-chromen-3-yl)methyl­idene]benzohydrazide monohydrate (Ishikawa & Watanabe, 2014*b* ▸), *N*-[(*E*)-(3-fluoro­pyridin-2-yl)methyl­idene]benzohydrazide monohydrate (Nair *et al.*, 2012 ▸), (*E*)-4-meth­oxy-*N*-(2,3,4-tri­meth­oxy­benzyl­idene)benzohydrazide monohydrate (Veer­am­anikandan *et al.*, 2016 ▸), 4-chloro-*N*-[(*E*)-2-chloro­benzyl­idene]benzohydrazide monohydrate (Mague *et al.*, 2014 ▸), 4-chloro-*N*-[(*Z*)-4-(di­methyl­amino)­benzyl­idene]benzo­hydra­zide monohydrate (Fun *et al.*, 2008 ▸), (*E*)-*N*-(4-but­oxy-3-meth­oxy­benzyl­idene)benzohydrazide (Zhen & Han, 2005 ▸) and (*E*)-4-hy­droxy-*N*-(3-hy­droxy­benzyl­idene)benzo­hydrazide monohydrate (Harrison *et al.*, 2014 ▸). The presence of O—H⋯N hydrogen bonds in addition to water-mediated O—H⋯O inter­actions is a common feature in many of the reported structures, but such an O—H⋯N inter­action is not observed in the title compound.

## Synthesis and crystallization   

Phosphoryl chloride (POCl_3_) (0.171mol) was slowly added to dry dimethyl formamide at 273 K, and then 3,4-di­hydro­naphthalen-1(2*H*)-one (0.174 mol) was added. The mixture was stirred at 353 K for 1.5 h. The reaction mixture was then poured into aqueous sodium acetate (3 mol l^−1^) and the product was extracted with ethyl acetate. Evaporating the ethyl acetate gave an oil, which on cooling solidified to yield 1-chloro-3,4-di­hydro­naphthalene-2-carbaldehyde. The title compound was prepared by refluxing 1-chloro-3,4-di­hydro­naphthalene-2-carbaldehyde (0.01 mol) with benzohydrazide (0.01 mol) in ethanol (5 ml) and few drops of acetic acid for 8 h. The reaction mixture was then cooled to room temperature, excess ethanol was removed under vacuum and the residue was quenched with ice. The precipitate was filtered, dried and crystallized from ethanol. The completion of the reaction was monitored by thin layer chromatography. Single crystals suitable for X-ray diffraction study were grown from an *N*,*N*-di­methyl­formamide solution by slow evaporation. Yield: 86%; m.p.: 438–440 K, colourless solid. ^1^H NMR (DMSO-*d*_6_, 400 MHz, ppm): *δ* 12.10 (*s*, 1H, NH), 8.77 (*s*, 1H), 7.87 (*d*, *J* = 7.2, 2H), 7.64–7.25 (*m*, 7H), 2.808–2.764 (*m*, 4H). ^13^C NMR: *δ* 163.34, 143.77, 144.5, 136.97, 132.72, 132.63, 131.65, 130.73, 129.80, 129.49, 128.16, 128.00, 127.41, 124.94, 29.50, 26.54, 23.65, 21.51. Mass calculated for C_18_H_15_ClN_2_O [*M*+H]^+^: 310.08; found: 310.9758.

## Refinement   

Crystal data, data collection and structure refinement details are summarized in Table 4 ▸. The N-bound H atom (H6) and water H atoms (H2*A* and H2*B*) were located in a difference-Fourier map and refined isotropically. All C-bound H atoms were placed in idealized positions (C—H = 0.93 or 0.97 Å) and treated as riding with *U*_iso_(H) = 1.2*U*_eq_(C).

## Supplementary Material

Crystal structure: contains datablock(s) global, I. DOI: 10.1107/S2056989019017183/is5528sup1.cif

Structure factors: contains datablock(s) I. DOI: 10.1107/S2056989019017183/is5528Isup2.hkl

CCDC reference: 1973816

Additional supporting information:  crystallographic information; 3D view; checkCIF report

## Figures and Tables

**Table 1:** Hydrogen-bond geometry (Å, °)

*D*—H⋯*A*	*D*—H	H⋯*A*	*D*⋯*A*	*D*—H⋯*A*
O2—H2*A*⋯O1^i^	0.78 (5)	2.06 (5)	2.829 (4)	166 (5)
O2—H2*B*⋯O1	0.96 (6)	1.90 (6)	2.858 (4)	171 (5)
N6—H6⋯O2^ii^	0.93 (5)	1.98 (5)	2.869 (4)	159 (4)
C1—H1⋯O2^ii^	0.93	2.47	3.350 (5)	158
C8—H8⋯O2^ii^	0.93	2.48	3.261 (5)	142

Symmetry codes: (i) 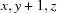; (ii) 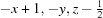.

**Table 2:** List of inter­molecular inter­action energies (kJ mol^−1^) in the crystal of the title compound

Code	Symmetry	Centroid distance	*E*_col_	*E*_pol_	*E*_energy-dispersive_	*E*_rep_	*E*_total_	Inter­action
*A*	*x*, *y* + 1, *z*	4.812	−37.4	−14.2	−64.3	88.7	−27.1	O—H⋯O
*B*	-*x* + 1, −*y* + 1, *z* − 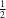	7.513	−6.7	−4.3	−20.9	15.9	−16.0	C—H⋯O
*C*	-*x* + 1, −*y* + 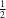 , *z*	9.210	−2.2	−4.2	−16.1	6.6	−15.9	Cl⋯H
*D*	-*x* + 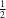 , *y* − 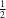 , *z* + 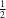	11.475	−1.9	−0.9	−8.9	3.8	−7.9	H⋯H
*E*	*x*, *y*, *z*	5.924	−33.1	−11.6	−12.3	33.1	−23.9	O—H⋯O
*F*	*x*, *y* − 1, *z*	4.077	−38.5	−12.0	−13.0	30.7	−32.8	N—H⋯O

**Table 3:** HUMO–LUMO energies and quantum-chemical parameters (eV) for the title compound

HOMO energy: *E*_H_	−5.7777
LUMO energy: *E*_L_	−2.1428
Energy gap: *E*_g_ = *E*_H_ − *E*_L_	3.6349
Chemical hardness: η = |*E*_H_ − *E*_L_|/2	1.8174
Softness: ζ = 1/2η	0.2751
Electrophilicity index: ω = μ^2^/2η	4.3148
Chemical Potential: μ = −(*E*_H_ + *E*_L_/2)	−3.9602
Electronegativity: χ = -μ	3.9602

**Table 4:** Experimental details

Crystal data	
Chemical formula	C_18_H_15_ClN_2_O·H_2_O
*M*_r_	328.78
Crystal system, space group	Orthorhombic, *P* *n* *a*2_1_
Temperature (K)	301
*a*, *b*, *c* (Å)	26.2059 (18), 4.8119 (3), 12.8084 (9)
*V* (Å^3^)	1615.14 (19)
*Z*	4
Radiation type	Mo *K*α
μ (mm^−1^)	0.25
Crystal size (mm)	0.28 × 0.22 × 0.21

Data collection	
Diffractometer	Bruker APEXII microsource
Absorption correction	Multi-scan (*SADABS*; Bruker, 2012 ▸)
*T*_min_, *T*_max_	0.890, 0.915
No. of measured, independent and observed [*I* > 2σ(*I*)] reflections	51179, 4917, 3162
*R*_int_	0.069
(sin θ/λ)_max_ (Å^−1^)	0.715

Refinement	
*R*[*F* ^2^ > 2σ(*F* ^2^)], *wR*(*F* ^2^), *S*	0.045, 0.151, 1.03
No. of reflections	4917
No. of parameters	220
No. of restraints	1
H-atom treatment	H atoms treated by a mixture of independent and constrained refinement
Δρ_max_, Δρ_min_ (e Å^−3^)	0.18, −0.30
Absolute structure	Flack *x* determined using 1242 quotients [(*I* ^+^)−(*I* ^−^)]/[(*I* ^+^)+(*I* ^−^)] (Parsons *et al.*, 2013 ▸)
Absolute structure parameter	−0.04 (2)

Computer programs: *APEX2* and *SAINT* (Bruker, 2012 ▸), *SIR2011* (Burla *et al.*, 2012 ▸), *SHELXL2018* (Sheldrick, 2015 ▸), *ORTEP-3 for Windows* and *WinGX* (Farrugia, 2012 ▸), *Mercury* (Macrae *et al.*, 2008 ▸), *PLATON* (Spek, 2009 ▸) and *publCIF* (Westrip, 2010 ▸).

## supplementary crystallographic information

### Crystal data

**Table:**

C_18_H_15_ClN_2_O·H_2_O	*F*(000) = 688
*M**_r_* = 328.78	*D*_x_ = 1.352 Mg m^−^^3^
Orthorhombic, *P**n**a*2_1_	Mo *K*α radiation, λ = 0.71073 Å
Hall symbol: P 2c -2n	Cell parameters from 689 reflections
*a* = 26.2059 (18) Å	θ = 2.2–30.1°
*b* = 4.8119 (3) Å	µ = 0.25 mm^−^^1^
*c* = 12.8084 (9) Å	*T* = 301 K
*V* = 1615.14 (19) Å^3^	Block, white
*Z* = 4	0.28 × 0.22 × 0.21 mm

### Data collection

**Table:**

Bruker APEXII microsource diffractometer	4917 independent reflections
Radiation source: microfocus sealed X-ray tube	3162 reflections with *I* > 2σ(*I*)
Mirror optics monochromator	*R*_int_ = 0.069
Detector resolution: 7.9 pixels mm^-1^	θ_max_ = 30.6°, θ_min_ = 2.2°
ω and φ scans	*h* = −37→36
Absorption correction: multi-scan (SADABS; Bruker, 2012)	*k* = −6→6
*T*_min_ = 0.890, *T*_max_ = 0.915	*l* = −18→18
51179 measured reflections	

### Refinement

**Table:**

Refinement on *F*^2^	Secondary atom site location: difference Fourier map
Least-squares matrix: full	Hydrogen site location: mixed
*R*[*F*^2^ > 2σ(*F*^2^)] = 0.045	H atoms treated by a mixture of independent and constrained refinement
*wR*(*F*^2^) = 0.151	*w* = 1/[σ^2^(*F*_o_^2^) + (0.0735*P*)^2^ + 0.2866*P*] where *P* = (*F*_o_^2^ + 2*F*_c_^2^)/3
*S* = 1.03	(Δ/σ)_max_ = 0.001
4917 reflections	Δρ_max_ = 0.18 e Å^−^^3^
220 parameters	Δρ_min_ = −0.30 e Å^−^^3^
1 restraint	Absolute structure: Flack *x* determined using 1242 quotients [(*I*^+^)-(*I*^-^)]/[(*I*^+^)+(*I*^-^)] (Parsons *et al.*, 2013)
Primary atom site location: structure-invariant direct methods	Absolute structure parameter: −0.04 (2)

### Special details

**Table:**

Geometry. All esds (except the esd in the dihedral angle between two l.s. planes) are estimated using the full covariance matrix. The cell esds are taken into account individually in the estimation of esds in distances, angles and torsion angles; correlations between esds in cell parameters are only used when they are defined by crystal symmetry. An approximate (isotropic) treatment of cell esds is used for estimating esds involving l.s. planes.

### Fractional atomic coordinates and isotropic or equivalent isotropic displacement parameters (Å^2^)

**Table:**

	*x*	*y*	*z*	*U*_iso_*/*U*_eq_	
Cl01	0.64431 (5)	0.3485 (3)	0.24491 (8)	0.0815 (4)	
O1	0.48097 (11)	−0.3470 (5)	0.58021 (18)	0.0577 (6)	
O2	0.48890 (14)	0.1565 (7)	0.6974 (2)	0.0676 (8)	
N6	0.50596 (11)	−0.2027 (6)	0.4205 (2)	0.0463 (6)	
N2	0.54124 (11)	−0.0257 (5)	0.4646 (2)	0.0469 (6)	
C1	0.44073 (17)	−0.6214 (7)	0.3284 (3)	0.0589 (9)	
H1	0.464242	−0.537211	0.284202	0.071*	
C2	0.40586 (19)	−0.8116 (8)	0.2890 (3)	0.0699 (11)	
H2	0.405810	−0.852699	0.218085	0.084*	
C3	0.37164 (16)	−0.9389 (9)	0.3536 (4)	0.0677 (11)	
H3	0.348710	−1.067883	0.326756	0.081*	
C4	0.37102 (15)	−0.8766 (9)	0.4583 (4)	0.0661 (10)	
H4	0.347389	−0.961795	0.501996	0.079*	
C5	0.40541 (13)	−0.6878 (8)	0.4987 (3)	0.0543 (8)	
H5	0.405023	−0.648210	0.569795	0.065*	
C6	0.44038 (12)	−0.5569 (6)	0.4347 (2)	0.0440 (7)	
C7	0.47695 (12)	−0.3606 (6)	0.4845 (2)	0.0429 (6)	
C8	0.57029 (13)	0.1000 (8)	0.3996 (3)	0.0514 (8)	
H8	0.566552	0.073299	0.328142	0.062*	
C9	0.60942 (13)	0.2864 (7)	0.4405 (3)	0.0500 (7)	
C10	0.61014 (14)	0.3515 (8)	0.5571 (3)	0.0546 (8)	
H10A	0.593193	0.203807	0.595282	0.066*	
H10B	0.591853	0.523334	0.570138	0.066*	
C11	0.66436 (15)	0.3790 (9)	0.5944 (3)	0.0603 (9)	
H11A	0.664204	0.450456	0.665242	0.072*	
H11B	0.679939	0.196212	0.595934	0.072*	
C12	0.69626 (13)	0.5672 (8)	0.5273 (3)	0.0548 (8)	
C13	0.73640 (15)	0.7232 (10)	0.5674 (4)	0.0703 (11)	
H13	0.743717	0.714146	0.638323	0.084*	
C14	0.76533 (16)	0.8898 (10)	0.5042 (5)	0.0796 (14)	
H14	0.791894	0.993296	0.532615	0.096*	
C15	0.75553 (16)	0.9049 (10)	0.4007 (5)	0.0771 (13)	
H15	0.775202	1.020496	0.358664	0.093*	
C16	0.71653 (16)	0.7501 (10)	0.3565 (4)	0.0692 (11)	
H16	0.710430	0.759136	0.285089	0.083*	
C17	0.68627 (13)	0.5795 (8)	0.4204 (3)	0.0549 (8)	
C18	0.64461 (13)	0.4061 (8)	0.3791 (3)	0.0536 (8)	
H2A	0.4899 (19)	0.281 (11)	0.658 (4)	0.068 (14)*	
H2B	0.4887 (19)	−0.005 (13)	0.653 (4)	0.082 (15)*	
H6	0.4993 (18)	−0.204 (10)	0.349 (4)	0.071 (13)*	

### Atomic displacement parameters (Å^2^)

**Table:**

	*U*^11^	*U*^22^	*U*^33^	*U*^12^	*U*^13^	*U*^23^
Cl01	0.0854 (7)	0.1131 (10)	0.0460 (5)	−0.0167 (6)	0.0052 (5)	0.0096 (6)
O1	0.0824 (17)	0.0516 (13)	0.0391 (11)	−0.0173 (12)	−0.0005 (11)	0.0002 (10)
O2	0.107 (2)	0.0580 (17)	0.0377 (12)	−0.0104 (15)	−0.0058 (13)	0.0033 (13)
N6	0.0548 (15)	0.0444 (14)	0.0396 (13)	−0.0079 (12)	−0.0029 (12)	0.0010 (11)
N2	0.0532 (15)	0.0418 (13)	0.0456 (13)	−0.0061 (11)	−0.0046 (11)	0.0014 (11)
C1	0.082 (2)	0.0491 (19)	0.0460 (18)	−0.0105 (17)	−0.0023 (17)	−0.0016 (15)
C2	0.099 (3)	0.056 (2)	0.054 (2)	−0.009 (2)	−0.017 (2)	−0.0074 (18)
C3	0.063 (2)	0.052 (2)	0.088 (3)	−0.0062 (18)	−0.016 (2)	−0.009 (2)
C4	0.051 (2)	0.063 (2)	0.085 (3)	−0.0085 (17)	0.0055 (19)	−0.004 (2)
C5	0.0508 (18)	0.0550 (19)	0.057 (2)	−0.0037 (15)	0.0072 (15)	−0.0048 (16)
C6	0.0511 (17)	0.0363 (13)	0.0445 (15)	0.0031 (12)	−0.0035 (13)	−0.0007 (12)
C7	0.0513 (16)	0.0380 (14)	0.0393 (15)	0.0017 (12)	−0.0007 (12)	0.0007 (12)
C8	0.0556 (18)	0.0532 (18)	0.0454 (17)	−0.0059 (15)	0.0009 (14)	0.0022 (14)
C9	0.0533 (18)	0.0493 (17)	0.0475 (18)	0.0011 (14)	0.0012 (14)	0.0040 (14)
C10	0.0507 (17)	0.064 (2)	0.0495 (18)	−0.0105 (16)	0.0004 (14)	−0.0084 (15)
C11	0.063 (2)	0.064 (2)	0.054 (2)	0.0048 (18)	−0.0070 (17)	0.0023 (17)
C12	0.0467 (17)	0.0496 (17)	0.068 (2)	0.0052 (14)	−0.0017 (16)	−0.0001 (17)
C13	0.055 (2)	0.073 (3)	0.083 (3)	−0.0009 (19)	−0.008 (2)	−0.008 (2)
C14	0.050 (2)	0.072 (3)	0.117 (5)	−0.0076 (19)	−0.003 (2)	−0.001 (3)
C15	0.056 (2)	0.069 (3)	0.107 (4)	−0.009 (2)	0.010 (2)	0.015 (2)
C16	0.062 (2)	0.069 (2)	0.077 (3)	0.0004 (19)	0.009 (2)	0.016 (2)
C17	0.0453 (17)	0.0497 (18)	0.070 (2)	0.0026 (14)	−0.0001 (15)	0.0058 (17)
C18	0.0548 (18)	0.0603 (19)	0.0457 (17)	−0.0032 (15)	0.0027 (15)	0.0074 (16)

### Geometric parameters (Å, º)

**Table:**

Cl01—C18	1.741 (4)	C8—H8	0.9300
O1—C7	1.233 (4)	C9—C18	1.342 (5)
O2—H2A	0.79 (6)	C9—C10	1.527 (5)
O2—H2B	0.96 (6)	C10—C11	1.505 (5)
N6—C7	1.352 (4)	C10—H10A	0.9700
N6—N2	1.378 (4)	C10—H10B	0.9700
N6—H6	0.93 (5)	C11—C12	1.502 (6)
N2—C8	1.280 (4)	C11—H11A	0.9700
C1—C2	1.389 (6)	C11—H11B	0.9700
C1—C6	1.396 (5)	C12—C13	1.390 (6)
C1—H1	0.9300	C12—C17	1.396 (5)
C2—C3	1.365 (7)	C13—C14	1.368 (7)
C2—H2	0.9300	C13—H13	0.9300
C3—C4	1.374 (7)	C14—C15	1.352 (7)
C3—H3	0.9300	C14—H14	0.9300
C4—C5	1.381 (5)	C15—C16	1.385 (7)
C4—H4	0.9300	C15—H15	0.9300
C5—C6	1.381 (5)	C16—C17	1.404 (6)
C5—H5	0.9300	C16—H16	0.9300
C6—C7	1.489 (4)	C17—C18	1.472 (5)
C8—C9	1.459 (5)		
			
H2A—O2—H2B	104 (5)	C9—C10—H10A	109.7
C7—N6—N2	118.4 (3)	C11—C10—H10A	109.7
C7—N6—H6	119 (3)	C9—C10—H10B	109.7
N2—N6—H6	122 (3)	C11—C10—H10B	109.7
C8—N2—N6	115.1 (3)	H10A—C10—H10B	108.2
C2—C1—C6	119.8 (4)	C12—C11—C10	113.4 (3)
C2—C1—H1	120.1	C12—C11—H11A	108.9
C6—C1—H1	120.1	C10—C11—H11A	108.9
C3—C2—C1	120.5 (4)	C12—C11—H11B	108.9
C3—C2—H2	119.7	C10—C11—H11B	108.9
C1—C2—H2	119.7	H11A—C11—H11B	107.7
C4—C3—C2	120.1 (4)	C13—C12—C17	118.7 (4)
C4—C3—H3	120.0	C13—C12—C11	122.4 (4)
C2—C3—H3	120.0	C17—C12—C11	118.9 (3)
C3—C4—C5	120.1 (4)	C14—C13—C12	121.2 (5)
C3—C4—H4	119.9	C14—C13—H13	119.4
C5—C4—H4	119.9	C12—C13—H13	119.4
C6—C5—C4	120.7 (4)	C13—C14—C15	120.4 (4)
C6—C5—H5	119.7	C13—C14—H14	119.8
C4—C5—H5	119.7	C15—C14—H14	119.8
C5—C6—C1	118.8 (3)	C14—C15—C16	120.8 (4)
C5—C6—C7	117.5 (3)	C14—C15—H15	119.6
C1—C6—C7	123.6 (3)	C16—C15—H15	119.6
O1—C7—N6	121.7 (3)	C15—C16—C17	119.6 (4)
O1—C7—C6	121.0 (3)	C15—C16—H16	120.2
N6—C7—C6	117.3 (3)	C17—C16—H16	120.2
N2—C8—C9	118.4 (3)	C16—C17—C12	119.4 (4)
N2—C8—H8	120.8	C16—C17—C18	122.8 (4)
C9—C8—H8	120.8	C12—C17—C18	117.9 (3)
C18—C9—C8	122.4 (3)	C9—C18—C17	122.8 (3)
C18—C9—C10	118.5 (3)	C9—C18—Cl01	120.5 (3)
C8—C9—C10	119.1 (3)	C17—C18—Cl01	116.6 (3)
C9—C10—C11	109.9 (3)		
			
C7—N6—N2—C8	174.4 (3)	C10—C11—C12—C13	149.3 (4)
C6—C1—C2—C3	−0.8 (6)	C10—C11—C12—C17	−32.7 (5)
C1—C2—C3—C4	0.9 (7)	C17—C12—C13—C14	1.0 (6)
C2—C3—C4—C5	−0.8 (6)	C11—C12—C13—C14	179.0 (4)
C3—C4—C5—C6	0.7 (6)	C12—C13—C14—C15	−0.4 (7)
C4—C5—C6—C1	−0.7 (5)	C13—C14—C15—C16	−0.7 (8)
C4—C5—C6—C7	−178.7 (3)	C14—C15—C16—C17	1.1 (7)
C2—C1—C6—C5	0.8 (5)	C15—C16—C17—C12	−0.5 (6)
C2—C1—C6—C7	178.6 (3)	C15—C16—C17—C18	−179.1 (4)
N2—N6—C7—O1	0.9 (5)	C13—C12—C17—C16	−0.6 (5)
N2—N6—C7—C6	−178.0 (3)	C11—C12—C17—C16	−178.7 (4)
C5—C6—C7—O1	10.7 (5)	C13—C12—C17—C18	178.2 (4)
C1—C6—C7—O1	−167.2 (3)	C11—C12—C17—C18	0.1 (5)
C5—C6—C7—N6	−170.5 (3)	C8—C9—C18—C17	−175.8 (3)
C1—C6—C7—N6	11.6 (5)	C10—C9—C18—C17	5.0 (5)
N6—N2—C8—C9	−178.7 (3)	C8—C9—C18—Cl01	1.3 (5)
N2—C8—C9—C18	173.5 (3)	C10—C9—C18—Cl01	−177.9 (3)
N2—C8—C9—C10	−7.3 (5)	C16—C17—C18—C9	−166.5 (4)
C18—C9—C10—C11	−36.7 (5)	C12—C17—C18—C9	14.8 (5)
C8—C9—C10—C11	144.1 (3)	C16—C17—C18—Cl01	16.3 (5)
C9—C10—C11—C12	49.2 (5)	C12—C17—C18—Cl01	−162.4 (3)

### Hydrogen-bond geometry (Å, º)

**Table:**

*D*—H···*A*	*D*—H	H···*A*	*D*···*A*	*D*—H···*A*
O2—H2*A*···O1^i^	0.78 (5)	2.06 (5)	2.829 (4)	166 (5)
O2—H2*B*···O1	0.96 (6)	1.90 (6)	2.858 (4)	171 (5)
N6—H6···O2^ii^	0.93 (5)	1.98 (5)	2.869 (4)	159 (4)
C1—H1···O2^ii^	0.93	2.47	3.350 (5)	158
C8—H8···O2^ii^	0.93	2.48	3.261 (5)	142

Symmetry codes: (i) *x*, *y*+1, *z*; (ii) −*x*+1, −*y*, *z*−1/2.
